# Sex differences in the diagnosis, treatment and prognosis of cancer: the rationale for an individualised approach

**DOI:** 10.1007/s12094-023-03112-w

**Published:** 2023-02-18

**Authors:** Ruth Vera, Oscar Juan-Vidal, María José Safont-Aguilera, Francisco Ayala de la Peña, Aránzazu González del Alba

**Affiliations:** 1 Department of Medical Oncology, University Hospital of Navarra, Pamplona. IdiSNA, Navarra’s Health Research Institute, Irunlarrea 3, 31190 Pamplona, Spain; 2 Department of Medical Oncology, University Hospital La Fe, Valencia, Spain; 3 Department of Medical Oncology, University General Hospital of Valencia, Valencia University, Valencia. CIBERONC, Valencia, Spain; 4 Medical Oncology, Department of Haematology and Oncology, University General Hospital Morales Meseguer, Murcia, Spain; 5 Genitourinary Tumour Unit, Department of Medical Oncology, University Hospital Puerta de Hierro, Majadahonda, Madrid, Spain

**Keywords:** Cancer, Epidemiology, Sex, Female, Male, Treatment

## Abstract

**Background:**

Precision medicine in oncology aims to identify the most beneficial interventions based on a patient’s individual features and disease. However, disparities exist when providing cancer care to patients based on an individual’s sex.

**Objective:**

To discuss how sex differences impact the epidemiology, pathophysiology, clinical manifestations, disease progression, and response to treatment, with a focus on data from Spain.

**Results:**

Genetic and environmental factors (social or economic inequalities, power imbalances, and discrimination) that contribute to these differences adversely affect cancer patient health outcomes. Increased health professional awareness of sex differences is essential to the success of translational research and clinical oncological care.

**Conclusions:**

The Sociedad Española de Oncología Médica created a Task Force group to raise oncologists’ awareness and to implement measures to address sex differences in cancer patient management in Spain. This is a necessary and fundamental step towards optimizing precision medicine that will benefit all individuals equally and equitably.

## Introduction

‘Precision medicine’ is defined as ‘*a healthcare approach with the primary aim of identifying which interventions are likely to be of most benefit to which patients based upon the features of the individual and their disease*’ [1]. Careful consideration of sex differences is a fundamental step towards precision medicine that will promote equality and equity in healthcare [2].

The terms ‘sex’ and ‘gender’ are not interchangeable. ‘Sex’ refers to the biological differences between males and females, and encompasses sex organs, endogenous hormones and chromosomes [3]. ‘Gender’, however, is a sociocultural construction that encompasses the roles, norms and behaviours expected for males and females in society, which may or may not correspond to their sex [3, 4]. Each individual’s health is determined by both their biological sex and gender expression [3] because access to healthcare and interactions with healthcare professionals can be influenced by sex and/or gender due to social or economic inequalities, power imbalances or discrimination [4, 5].

Oncology research has mainly focussed on the genomic profile of a cancer to personalise treatment, and current approaches to precision medicine in oncology generally do not include factors such as sex or gender in therapeutic decisions [6]. It is of increasing concern that sex and gender influence cancer susceptibility, progression, survival and response to different treatments; as such, there is growing recognition that a patient’s sex and gender also need to be considered in the formulation of an optimal treatment approach [7].

There is evidence to suggest that women do not receive the same treatment for cancer as men [8, 9]. This is unsurprising because women have been historically excluded from clinical trials for various reasons, resulting in research and medical attention focussed on male physiology; indeed, the diagnosis, treatment, and prevention of disease originates from studies carried out mainly on male cells, male mice and men [10]. The Sociedad Española de Oncología Médica (SEOM) in Spain has created a Women’s Task Force, named Oncogenyx, to analyse the impact of sex and gender on the diagnosis, treatment and outcomes of cancer patients. The aim is to improve the quality of care for cancer patients in Spain by implementing appropriate measures to address sex/gender disparities. One of the first initiatives of Oncogenyx was to carry out a survey among SEOM members to assess the awareness of Spanish oncologists with regard to sex differences in the diagnosis, treatment and prognosis of patients with cancer. Participation in the survey was not very high, which indicates the dire need to inform and educate oncologists on these sex differences. This article describes the rationale for the SEOM Task Force by discussing how sex differences impact the diagnosis, treatment and outcomes of cancer, with a focus on data from Spain.

### Sex differences in cancer incidence and mortality

**Fig. 1:**
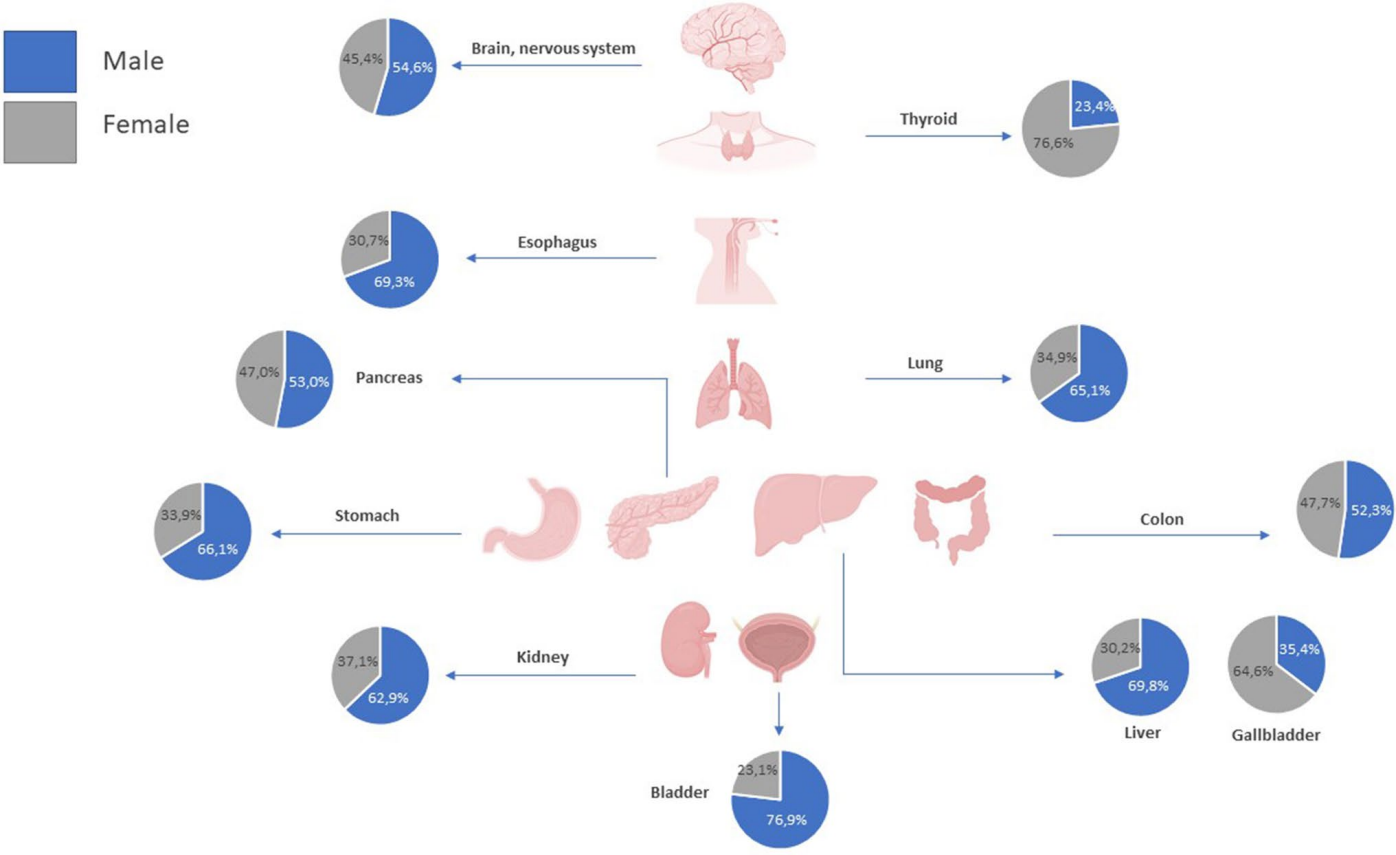
Sexual dimorphism in the incidence of different cancer types unrelated to reproductive functions representing the percentages of new diagnosed cancer cases in 2020 among men and women. The percentage values have been calculated using data retrieved from the Global Cancer Observatory GLOBOCAN 2020 [68]. The data have been extracted from Cardano M [12]. Created using Biorender

Disparities occur in cancer incidence and mortality based on a patient’s sex [11]. Although women in Europe tend to report worse general health than men, the probability of somatic tumour development is higher and the prognosis is worse in men (Fig. 1) [12]. Overall, the age-standardised incidence and mortality rates of patients with cancer are higher in men than in women, both globally [13] and in Spain [14, 15]. The major exceptions (excluding cancers specifically related to reproductive organs, such as breast cancer or prostate cancer) are thyroid and gallbladder cancer (Fig. 1), both of which occur at higher rates in women than in men [12–14].

In Spain, the incidence of lung cancer is lower in women than in men [16–18], but the difference between the sexes is becoming less marked as a result of changes in smoking habits in men and women [16, 17]. Consequently, the incidence of lung cancer has somewhat stabilised in men, while it continues to increase in women [16]. Spanish women also show lower age-adjusted rates of mortality compared with Spanish men across a range of cancers, including colorectal cancer [19, 20], cancer of the lip, oral cavity or pharynx [21], lung cancer [22–24], non-melanoma skin cancer [25], oesophageal cancer [26] and pancreatic cancer [27, 28].

With regard to sex-specific tumours, the leading causes of premature mortality among women and men are breast cancer and prostate cancer, respectively [29].

### Potential reasons for sex differences

Cancer occurs as a result of a complex interplay between genetic and environmental factors, which differs between the sexes.

### Genetic factors

The Cancer Genome Atlas (TCGA) analysed the molecular profiles of a range of cancers in males and females and identified those with strong or weak sex-related differences (Table 1) [30]. The tumour mutational burden tends to be lower in females than males in various cancers [31, 32]; this may affect the antigenicity of the tumour and therefore the efficacy of immune checkpoint inhibitor (ICI) therapy [33]. In addition, the X and Y chromosomes themselves may play a role in determining cancer biology [11, 34]. Tumour suppressor genes may be present on the inactive X chromosome (Xi) in females, and genes called ‘escape from typical X-inactivation tumour suppressors’ (EXITS) can confer protection against cancer in females that is not present in males [34]. Similarly, men may develop extreme downregulation or loss of Y chromosome expression, which increases their risk of cancer through the loss of tumour suppressor genes on the Y chromosome [35]. There is also evidence of differences between the sexes in response to genotoxic stress and activation of DNA damage repair pathways, with women expressing higher levels of DNA repair genes and acquiring fewer somatic mutations over a lifetime, than men [12].

**Table 1:** Cancers showing strong or weak sex-related molecular differences [30], based on somatic mutations, somatic copy number alterations, DNA methylation, mRNA expression, micro-RNA expression and protein expression

Weak sex-related differences	Strong sex-related differences
Lower grade brain glioma Glioblastoma multiforme Colon adenocarcinoma Rectal carcinoma Acute myeloid leukaemia	Thyroid carcinoma Head and neck squamous cell carcinoma Lung squamous cell carcinoma Lung adenocarcinoma Hepatocellular carcinoma Bladder urothelial carcinoma Papillary renal cell carcinoma Clear cell renal cell carcinoma

### Environmental factors

Socioeconomic inequity can impact on cancer occurrence and diagnosis in a number of ways, by affecting lifestyle behaviours, diet, smoking, alcohol consumption, awareness of risks, exposure to environmental pollutants and access to healthcare [36]. In Spain, social inequity has been associated with an increased risk of cancer incidence and mortality in both sexes, but the effect of socioeconomic deprivation on this risk varies between sexes and between cancer types [37, 38]. While inequalities between the sexes is not as marked in Spain as it is in some other countries, women still lag behind men in work opportunities, pay and educational attainment, and are still required to undertake more of the domestic, child-rearing and caregiving activities [29], with all the socioeconomic and lifestyle impacts these differences may confer.

### Sex differences in the pathophysiology of cancer

Biological sex is first and foremost a genetic modifier of disease pathophysiology, clinical presentation and response to treatment [2]. Sex hormones have different effects on the tumour microenvironment (TME), affecting the function of cancer-associated fibroblasts, the remodelling of the extracellular matrix, angiogenesis and possibly lymphangiogenesis [39]. Across a range of cancers, strong sex-related differences in the TME have been noted in relation to the profile of infiltrating immune cells, immune checkpoint gene expression and functional pathways [32, 40]. Moreover, the sex-related patterns of immune features differ by cancer type (e.g. between lung cancer and melanoma) [41]. Sex-related differences in cancer pathophysiology may explain why there are often differences between men and women in the predominant histological subtype or the stage of cancer at presentation [42, 43]. For example, in Spain, women with lung cancer present with more advanced disease compared with men [44, 45] and are less likely than men to have squamous cell carcinoma [45].

### Impact of sex differences on diagnosis and treatment

Although sex disparity in cancer incidence, aggressiveness and disease prognosis has been observed for a variety of cancers, relatively little is known and evaluated about the impact of sex on diagnosis and clinical disease management. A study in the US showed that women with pancreatic cancer had a longer time between symptom onset and diagnosis, and from diagnosis to surgery, compared with male patients [46]. Data from Spain show that there are sex-related differences in the time between screening or symptoms and diagnosis of rectal cancer, and that this form of cancer is suspected and confirmed earlier in men than it is in women [9]. As a result, women are more likely than men to be diagnosed later with disseminated disease [9]. An Italian study found that although greater adherence to colorectal screening programmes were by women, the sensitivity of screening was higher for men than women (80.1% vs 74.8%) [47]. Similarly, other inequalities may exist, such as a later diagnosis of, although rare, breast cancer in males [48].

The impact of sex differences on screening and diagnosis of cancer emphasises the importance of understanding the influences of sex differences across the cancer care continuum [8]. As diagnostic modalities become more automated in future, it is important to ensure that computer-assisted diagnostic tools using artificial intelligence (AI) do not introduce a sex bias in diagnoses, particularly if the AI training was based on an unequal number of images from males and females [49].

Awareness of sex differences across the cancer care continuum also extends to patient mental health: evidence from Spain indicates that women with cancer experience more anxiety than their male counterparts [50], highlighting the need to consider sex disparities in the management of the mental health of patients with cancer.

### Sex differences in the pharmacology of anticancer drugs

Most anticancer agents are administered at standard dosages according to body weight or body surface area, which may vary considerably between men and women [51]. Body surface area has been identified as an inaccurate method to calculate chemotherapy doses; the associated risk of underdosing was recognised over a decade ago [51]. Intrinsic sex-based differences in body weight, plasma volume, gastric emptying time, plasma protein levels, cytochrome P450 (CYP450) activity, drug transporter function and excretion activity influence the four major factors that contribute to pharmacokinetic variability in individuals (bioavailability, distribution, metabolism and elimination) [52]. For example, women have a larger distribution volume of lipophilic drugs, whereas men have a larger distribution volume of water-soluble drugs [52]. Men typically tend to have increased activity of CYP1A2, CYP2D6 and CYP2E1 enzymes, resulting in increased metabolism of their corresponding drug substrates, while women show higher CYP3A4 activity, which is integral in metabolising the majority of drugs [52]. These sex disparities affect the pharmacokinetic profile of a large number of anticancer drugs and are responsible for 20% overexposure in women [53].

### Impact of sex differences on response to treatment

Sex differences in metabolism and immune response may contribute to differential responses to treatment between men and women. As described above, women are less likely than men to respond to ICI therapy in a number of cancer types including non-small cell lung cancer [54] and melanoma [55], although this is not a universal finding [56]. In addition, male and female patients with similar genomic profiles may have a different response to treatment, and genomic biomarkers may be predictive in one sex but not the other. For example, in melanoma patients, the presence of *CFH*, *DGKG* or *PPP6C* mutations was predictive of a better response to ICI therapy in males but not in females [57]. These gene mutations were also significant predictors of response in the overall group [57], so unless researchers are aware of the potential for sex differences in predictive biomarkers, they may mistakenly believe that a biomarker that is predictive in men is also predictive in women (or vice versa).

### Impact of sex differences on cancer treatment outcomes

Sex differences in response to treatment contribute to different cancer outcomes between men and women. This has been shown in Spain where the female sex is associated with improved survival across a range of cancers [58], including oropharyngeal cancer (despite a similar rate of recurrence) [59] and bladder cancer [60]. In contrast, a US study reported a significantly higher 90-day mortality rate in women compared with men, despite similar use of optimal treatments for muscle-invasive bladder cancer in both sexes [61]. However, women in Spain have higher rates of temporary or permanent cancer-related disability compared with men [62], indicating that while men may die more readily from cancer, the burden of cancer among survivors is greater among females.

### Impact of sex differences on treatment safety and tolerability

A number of large-scale studies have shown that women are more likely to experience adverse events (AEs) during cancer treatment than men [63, 64]. Among 34,640 patients in the Adjuvant Colon Cancer End Points (ACCENT) database, the only AE that occurred significantly more often in men was transaminitis during treatment with capecitabine plus oxaliplatin [64]. In contrast, women experienced neutropenia, leukopenia, nausea and vomiting significantly more often than men, irrespective of the chemotherapy regimen they received [64]. Further, according to an analysis of data from clinical trials by the South Western Oncology Group Network, the risk of women developing severe AEs was 34% higher than men, specifically in the treatment domains of chemotherapy (74% vs 68%), immunotherapy (57% vs 49%) and targeted therapy (50% vs 45%) [63].

### Sex disparities in clinical research

Historically, biomedical research has focussed on male physiology, at all levels: basic, preclinical and clinical [65]. Biomedical research in some medical specialities, such as cardiology, already reflects the importance of sex differences as modulators of disease biology [53]. However, in oncology, the importance of these difference is underestimated. For example, there is evidence that women are under-represented in clinical trials of treatments for many different types of cancer. As a result, drugs are being approved based on research that was conducted principally in men [66], with the results of this research, including drug toxicity or efficacy, extrapolated to all patients, assuming similar biological behaviour. There is a risk that negative results of clinical studies conducted mainly in men may lead to a discontinuation of drug development for treatments that may be effective and well tolerated in women [65]. Moreover, female researchers are under-represented in oncology publications [67].

## Conclusion

There is growing evidence that sex differences influence cancer prevention, susceptibility, progression, survival and response to different treatments. The impact of biological sex on the aetiology of cancer has not been fully elucidated, but there is clear evidence that the disease is not the same in men and women. Sex differences in cancer biology and treatment deserve more attention and systematic research that is equally representative of women and men. Interventional clinical trials evaluating sex-specific dosing regimens are needed to improve the balance between efficacy and toxicity of anticancer drugs. Clinicians’ increased awareness of sex differences in the epidemiology, pathophysiology, clinical manifestations, psychological effects, disease progression and response to treatment is essential to the success of oncological care and translational science. The SEOM has created a Task Force group to address sex differences in cancer biology and treatment, and to raise awareness of these differences among oncology professionals. The SEOM considers that the inclusion of a sex perspective is a necessary and fundamental step towards precision medicine that will benefit all individuals equally and equitably.

## Data Availability

The data generated or analysed are included in this article.

## References

[1] Yates LR, Seoane J, Le Tourneau C, Siu LL, Marais R, Michiels S (2018). The European Society for Medical Oncology (ESMO) precision medicine glossary. Ann Oncol.

[2] Mauvais-Jarvis F, Bairey Merz N, Barnes PJ, Brinton RD, Carrero JJ, DeMeo DL (2020). Sex and gender: modifiers of health, disease, and medicine. Lancet.

[3] Office of Research on Women's Health. Sex & gender. 2022. https://orwh.od.nih.gov/sex-gender#:~:text=%22Sex%22%20refers%20to%20biological%20differences,across%20societies%20and%20over%20time. Accessed July 12, 2022.

[4] World Health Organization. Gender and health. 2022. https://www.who.int/health-topics/gender#tab=tab_1. Accessed July 12, 2022.

[5] Freijomil-Vázquez C, Gastaldo D, Coronado C, Movilla-Fernández MJ (2021). Asymmetric power relations in gynaecological consultations for cervical cancer prevention: biomedical and gender issues. Int J Environ Res Pub Health.

[6] Gambardella V, Tarazona N, Cejalvo JM, Lombardi P, Huerta M, Roselló S (2020). Personalized medicine: recent progress in cancer therapy. Cancers (Basel).

[7] Özdemir BC, Oertelt-Prigione S, Adjei AA, Borchmann S, Haanen JB, Letsch A (2022). Investigation of sex and gender differences in oncology gains momentum: ESMO announces the launch of a Gender Medicine Task Force. Ann Oncol.

[8] Dijksterhuis WPM, Kalff MC, Wagner AD, Verhoeven RBH, Lemmens VEPP, Van Oijen MGH (2021). Gender differences in treatment allocation and survival of advanced gastroesophageal cancer: a population-based study. J Natl Cancer Inst.

[9] Sarasqueta C, Zunzunegui MV, Enriquez Navascues JM, Querejeta A, Placer C, Perales A (2020). Gender differences in stage at diagnosis and preoperative radiotherapy in patients with rectal cancer. BMC Cancer.

[10] Clayton JA (2016). Studying both sexes: a guiding principle for biomedicine. FASEB J.

[11] Lopes-Ramos CM, Quackenbush J, DeMeo DL (2020). Genome-wide sex and gender differences in cancer. Front Oncol.

[12] Cardano M, Buscemi G, Zannini L (2022). Sex disparities in DNA damage response pathways: novel determinants in cancer formation and therapy. iScience.

[13] Global Burden of Disease Cancer Collaboration (2018). Global, regional, and national cancer incidence, mortality, years of life lost, years lived with disability, and disability-adjusted life-years for 29 cancer groups, 1990 to 2016: a systematic analysis for the Global Burden of Disease Study. JAMA Oncol.

[14] Galceran J, Ameijide A, Carulla M, Mateso A, Quirós JR, Rojas D (2017). Cancer incidence in Spain, 2015. Clin Transl Oncol.

[15] Contiero P, Tagliabue G, Gatta G, Galceran J, Bulliard JL, Bertoldi M (2021). Variation of cancer incidence between and within GRELL countries. Int J Environ Res Pub Health.

[16] Guarga L, Ameijide A, Marcos-Gragera R, Carulla M, Delgadillo J, Borràs JM (2021). Trends in lung cancer incidence by age, sex and histology from 2012 to 2025 in Catalonia (Spain). Sci Rep.

[17] Redondo-Sánchez D, Marcos-Gragera R, Carulla M, Lopez A, de Munain C, Gregori S, Chillarón RJ (2021). Lung, breast and colorectal cancer incidence by socioeconomic status in Spain: a population-based multilevel study. Cancers (Basel).

[18] Zhang Y, Luo G, Etxeberria J, Hao Y (2021). Global patterns and trends in lung cancer incidence: a population-based study. J Thorac Oncol.

[19] Cayuela L, Rodríguez-Domínguez S, Giráldez Á, Cayuela A (2021). Regional differences in colorectal cancer mortality trends, Spain (1980–2018). Rev Esp Enferm Dig.

[20] Luque-Fernandez MA, Redondo-Sánchez D, Rodríguez-Barranco M, Chang-Chan YL, Salamanca-Fernández E, Núñez O (2020). Socioeconomic inequalities in colorectal cancer survival in Southern Spain: a multilevel population-based cohort study. Clin Epidemiol.

[21] Retegui G, Etxeberria J, Ugarte MD (2021). Estimating LOCP cancer mortality rates in small domains in Spain using its relationship with lung cancer. Sci Rep.

[22] Cayuela L, López-Campos JL, Otero R, Rodriguez Portal JA, Rodríguez-Domínguez S, Cayuela A (2021). The beginning of the trend change in lung cancer mortality trends in Spain, 1980–2018. Arch Bronconeumol (Engl Ed).

[23] Martín-Sánchez JC, Clèries R, Lidón-Moyano C, González-de Paz L, Martínez-Sánchez JM (2016). Differences between men and women in time trends in lung cancer mortality in Spain (1980–2013). Arch Bronconeumol.

[24] Moryson W, Stawinska-Witoszynska B (2021). Excess mortality of males due to malignant lung cancer in OECD countries. Int J Environ Res Pub Health.

[25] Sendín-Martin M, Hernández-Rodríguez JC, Durán-Romero AJ, Ortiz-Álvarez J, Conejo-Mir J, Pereyra-Rodríguez JJ (2021). Non-melanoma skin cancer mortality in Spain: a predictive model up to 2044. J Clin Med.

[26] Huang J, Koulaouzidis A, Marlicz W, Lok V, Chu C, Ho Ngai C (2021). Global burden, risk factors, and trends of esophageal cancer: an analysis of cancer registries from 48 countries. Cancers (Basel).

[27] Etxeberria J, Goicoa T, López-Abente G, Riebler A, Ugarte MD (2017). Spatial gender-age-period-cohort analysis of pancreatic cancer mortality in Spain (1990–2013). PLoS ONE.

[28] Seoane-Mato D, Nuñez O, Fernández-de-Larrea N, Pérez-Gómez B, Pollán M, López-Abente G (2018). Long-term trends in pancreatic cancer mortality in Spain (1952–2012). BMC Cancer.

[29] European Institute for Gender Equality. Gender Equality Index: Spain. 2020. https://eige.europa.eu/gender-equality-index/2020/country/ES. Accessed July 15, 2022.

[30] Yuan Y, Liu L, Chen H, Wang Y, Xu Y, Mao H (2016). Comprehensive characterization of molecular differences in cancer between male and female patients. Cancer Cell.

[31] Castro A, Pyke RM, Zhang X, Thompson WK, Day CP, Alexandrov LB (2020). Strength of immune selection in tumors varies with sex and age. Nat Commun.

[32] Han J, Yang Y, Li X, Wu J, Sheng Y, Qiu J (2022). Pan-cancer analysis reveals sex-specific signatures in the tumor microenvironment. Mol Oncol.

[33] Lee J, Kay K, Troike K, Ahluwalia MS, Lathia JD (2022). Sex differences in glioblastoma immunotherapy response. Neuromolecular Med.

[34] Haupt S, Caramia F, Klein SL, Rubin JB, Haupt Y (2021). Sex disparities matter in cancer development and therapy. Nat Rev Cancer.

[35] Cáceres A, Jene A, Esko T, Pérez-Jurado LA, González JR (2020). Extreme downregulation of chromosome Y and cancer risk in men. J Natl Cancer Inst.

[36] Afshar N, English DR, Milne RL (2021). Factors explaining socio-economic inequalities in cancer survival: a systematic review. Cancer Control.

[37] Santos-Sánchez V, Córdoba-Doña JA, Viciana F, Escolar-Pujolar A, Pozzi L, Ramis R (2020). Geographical variations in cancer mortality and social inequalities in southern Spain (Andalusia) 2002–2013. PLoS ONE.

[38] Santos-Sánchez V, Córdoba-Doña JA, García-Pérez J, Escolar-Pujolar A, Pozzi L, Ramis R (2020). Cancer mortality and deprivation in the proximity of polluting industrial facilities in an industrial region of Spain. Int J Environ Res Pub Health.

[39] Wuidar V, Gillot L, Dias Da Silva I, Lebeau A, Gallez A, Pequeux C (2021). Sex-based differences in the tumor microenvironment. Adv Exp Med Biol.

[40] Conforti F, Pala L, Pagan E, Bagnardi V, De Pas T, Queirolo P (2021). Sex-based dimorphism of anticancer immune response and molecular mechanisms of immune evasion. Clin Cancer Res.

[41] Ye Y, Jing Y, Li L, Mills GB, Diao L, Liu H (2020). Sex-associated molecular differences for cancer immunotherapy. Nat Commun.

[42] Choi Y, Kim N, Kim KW, Jo HH, Park J, Yoon H (2022). Sex-based differences in histology, staging, and prognosis among 2983 gastric cancer surgery patients. World J Gastroenterol.

[43] Kalff MC, Wagner AD, Verhoeven RHA, Lemmens VEPP, Van Laarhoven HWM, Gisbertz SS (2022). Sex differences in tumor characteristics, treatment, and outcomes of gastric and esophageal cancer surgery: nationwide cohort data from the Dutch Upper GI Cancer Audit. Gastric Cancer.

[44] Provencio M, Carcereny E, Rodríguez-Abreu D, Lópz-Castro R, Guirado M, Camps C (2019). Lung cancer in Spain: information from the Thoracic Tumors Registry (TTR study). Transl Lung Cancer Res.

[45] Ruano-Ravina A, Provencio M, Calvo de Juan V, Carcereny E, Estival A, Rodríguez-Abreu D (2021). Are there differences by sex in lung cancer characteristics at diagnosis? -a nationwide study. Transl Lung Cancer Res.

[46] Azap RA, Hyer JM, Diaz A, Tsilimigras DI, Mirdad RS, Pawlik TM (2021). Sex-based differences in time to surgical care among pancreatic cancer patients: a national study of Medicare beneficiaries. J Surg Oncol.

[47] Zorzi M, Fedato C, Grazzini G, Stocco FC, Banovich F, Bortoli A (2011). High sensitivity of five colorectal screening programmes with faecal immunochemical test in the Veneto Region Italy. Gut.

[48] de Blok CJM, Wiepjes CM, Nota NM, Van Engelen K, Adank MA, Dreijerink KMA (2019). Breast cancer risk in transgender people receiving hormone treatment: nationwide cohort study in the Netherlands. BMJ.

[49] Sies K, Winkler JK, Fink C, Bardehle F, Toberer F, Buhl T (2022). Does sex matter? Analysis of sex-related differences in the diagnostic performance of a market-approved convolutional neural network for skin cancer detection. Eur J Cancer.

[50] Parás-Bravo P, Paz-Zulueta M, Boixadera-Planas E, Fradejas Sastre V, Palacios-Ceña D, Fernández-de-las-Peñas C (2020). Cancer patients and anxiety: a gender perspective. Int J Environ Res Pub Health.

[51] Gurney H (2002). How to calculate the dose of chemotherapy. Br J Cancer.

[52] Gandhi M, Aweeka F, Greenblatt RM, Blaschke TF (2004). Sex differences in pharmacokinetics and pharmacodynamics. Annu Rev Pharmacol Toxicol.

[53] Wagner AD, Oertelt-Prigione S, Adjei A, Buclin T, Cristina V, Csajka C (2019). Gender medicine and oncology: report and consensus of an ESMO workshop. Ann Oncol.

[54] Conforti F, Pala L, Pagan E, Corti C, Bagnardi V, Queirolo P (2021). Sex-based differences in response to anti-PD-1 or PD-L1 treatment in patients with non-small-cell lung cancer expressing high PD-L1 levels A systematic review and meta-analysis of randomized clinical trials. ESMO Open.

[55] Jang SR, Nikita N, Banks J, Keith SW, Johnson JM, Wilson M (2021). Association between sex and immune checkpoint inhibitor outcomes for patients with melanoma. JAMA Netw Open.

[56] Yang F, Markovic SN, Molina JR, Halfdanarson TR, Pagliaro LC, Chintakuntlawar AV (2020). Association of sex, age, and eastern cooperative oncology group performance status with survival benefit of cancer immunotherapy in randomized clinical trials: a systematic review and meta-analysis. JAMA Netw Open.

[57] Shi F, Zhang W, Yang Y, Yang Y, Zhao J, Xie M (2021). Sex disparities of genomic determinants in response to immune checkpoint inhibitors in melanoma. Front Immunol.

[58] Chirlaque MD, Salmerón D, Galceran J, Ameijide A, Mateos A, Torrella A (2018). Cancer survival in adult patients in Spain Results from nine population-based cancer registries. Clin Transl Oncol.

[59] Anantharaman D, Billot A, Waterboer T, Gheit T, Abedi-Ardekani B, Lagiou P (2018). Predictors of oropharyngeal cancer survival in Europe. Oral Oncol.

[60] Ripoll J, Ramos M, Montaño J, Pons J, Ameijide A, Franch P (2021). Cancer-specific survival by stage of bladder cancer and factors collected by Mallorca Cancer Registry associated to survival. BMC Cancer.

[61] Marinaro J, Zeymo A, Egan J, Carvalho F, Krasnow R, Stamatakis L (2021). Sex and racial disparities in the treatment and outcomes of muscle-invasive bladder cancer. Urology.

[62] Badía X, Tort M, Manganelli AG, Camps C, Díaz-Rubio E (2019). The burden of cancer in Spain. Clin Transl Oncol.

[63] Unger JM, Vaidya R, Albain KS, LeBlanc M, Minasian LM, Gotay CC (2022). Sex differences in risk of severe adverse events in patients receiving immunotherapy, targeted therapy, or chemotherapy in cancer clinical trials. J Clin Oncol.

[64] Wagner AD, Grothey A, Andre T, Dixon JG, Wolmark N, Haller DG (2021). Sex and adverse events of adjuvant chemotherapy in colon cancer: an analysis of 34 640 patients in the ACCENT database. J Natl Cancer Inst.

[65] Karp NA, Katial R, Thacker K (2020). Sex in studies: the first level of personalisation. Physiol News.

[66] Dymanus KA, Butaney M, Magee DE, Hird AE, Luckenbaugh AN, Ma MW (2021). Assessment of gender representation in clinical trials leading to FDA approval for oncology therapeutics between 2014 and 2019: a systematic review-based cohort study. Cancer.

[67] González-Alvarez J, Sos-Peña R (2020). Women in contemporary cancer research. Int J Cancer.

[68] Sung H, Ferlay J, Siegel RL, Laversanne M, Soerjomataram I, Jemal A (2021). Global Cancer Statistics 2020: GLOBOCAN estimates of incidence and mortality worldwide for 36 cancers in 185 countries. CA Cancer J Clin.

